# XANES, EXAFS, Voltammetric, and Microhardness Studies of Manganese Dioxide–Lead–Lead Dioxide Vitroceramics

**DOI:** 10.3390/ma15196522

**Published:** 2022-09-20

**Authors:** S. Rada, M. Unguresan, Jing Zhang

**Affiliations:** 1 Physics and Chemistry Department, Technical University of Cluj-Napoca, 400020 Cluj-Napoca, Romania; 2 National Institute for Research and Development of Isotopic and Molecular Technologies, 400293 Cluj-Napoca, Romania; 3 Institute of High Energy Physics, Chinese Academy of Sciences, Beijing 100049, China

**Keywords:** MnO_2_-Pb-PbO_2_, XRD, XAS, cyclic voltammetry, Vickers

## Abstract

In this work we investigated the electrochemical performances and mechanical behavior of the manganese dioxide–lead dioxide–lead vitreous system. The structural and electrochemical properties of vitroceramics were investigated by the analysis of X-ray diffraction (XRD) spectra, X-ray absorption spectroscopy (XAS), and measurements of cyclic voltammetry and linear sweep voltammetry. The mechanical properties of the studied samples were determined by Vickers hardness values using the indentation method. The analysis of X-ray absorption near edge structure (XANES) data for the L3 edge of the lead and the radial distribution function data for the PbO_2_ and Pb model indicate the modification of Pb-O interatomic distances by the doping process. The voltammetric study reveals the vitroceramic with x = 90 mol% Pb as the most suitable for application as a lead acid battery electrode.

## 1. Introduction

Lead glasses have lower melting and glass transition temperatures and high refractive indices with low crystallization tendency. Until now, most studies discussed the effect of metal oxide levels in binary PbO_2_-Pb glasses and vitroceramics [1,2,3,4]. The previous studies [5] indicate that doping the PbO_2_-Pb vitreous system with MnO_2_ leads to improved electrochemical performance. In addition, the MnO_2_ content is responsible for the minimization of hydrogen evolution and passivation processes in the potential range from 0 to 2 V [6]. The main disadvantage is the weak reversibility of the cyclic voltammetry after several charge–discharge cycles due to the dimerization of the sulfate ions. Increases in the H_2_SO_4_ concentration and the discharge speed of the car battery after several charge/discharge cycles were identified.

In glasses containing lead oxide, PbO has dual nature because it can act as network former and modifier [7,8]. The effect of Pb on the network structures and how it affects the glass and glass ceramics properties are not yet reported [9].

The present study provides electrochemical and mechanical information on the MnO_2_-Pb-PbO_2_-based glasses—ceramics doped with 15 mol% MnO_2_. The influence of the lead content on the structure are investigated by XRD and XAS data. The electrochemical performances and values of Vickers hardness of the electrode materials after aging in 5 M sulphuric acid solution were evaluated.

## 2. Experimental Procedure

Samples with the chemical formula 15MnO_2_·85[(100 − x)PbO_2_·xPb] where x = 0–100 mol% Pb were synthesized using laboratory reagents as raw materials: MnO_2_, PbO_2_, and fine metallic lead powder. The mixtures of substances weighted in suitable proportions were homogenized in an agate mortar and introduced to alumina crucibles. The crucibles containing the mix were placed for 10 min in an electric oven fixed at 850 °C for 10 min. The melt was overturned directly at room temperature on a steel plate.

X-ray diffractograms were obtained with a diffractometer using a graphite monochromator for a copper anode tube (wavelength, λ = 1.54 Å). Fine powder samples were used to obtain the diffractograms.

The XAS measurements were recorded on the 4W1B installation. The resolution of the X-ray beam was about 1–3 eV at a radiation energy of 10 KeV at room temperature. XANES measurements in fluorescence mode were realized using X-ray energy of incident flux situated between 13,000 and 13,100 eV. For lead, the L3—edge is situated at 13,035 eV.

The voltammetric investigations were performed using an AUTOLAB PGSTAT 302N type (Metrohm Autolab, Utrecht, The Netherlands) potentiostat/galvanostat equipped with NOVA 1.11 software and an electrochemical cell with three electrodes. Prepared samples were used as working electrode. The calomel electrode and platinum were used as reference and counter electrode, respectively. H_2_SO_4_ of 5 M concentration was introduced in the cell as electrolyte solution.

The measurements of Vickers hardness were performed using a Nova microdurimeter equipped with a microscope and software. The penetrator was applied with a load of 0.3 kgf for 15 s.

## 3. Results and Discussions

### 3.1. Structural Investigation by XRD Data

**Figure 1:**
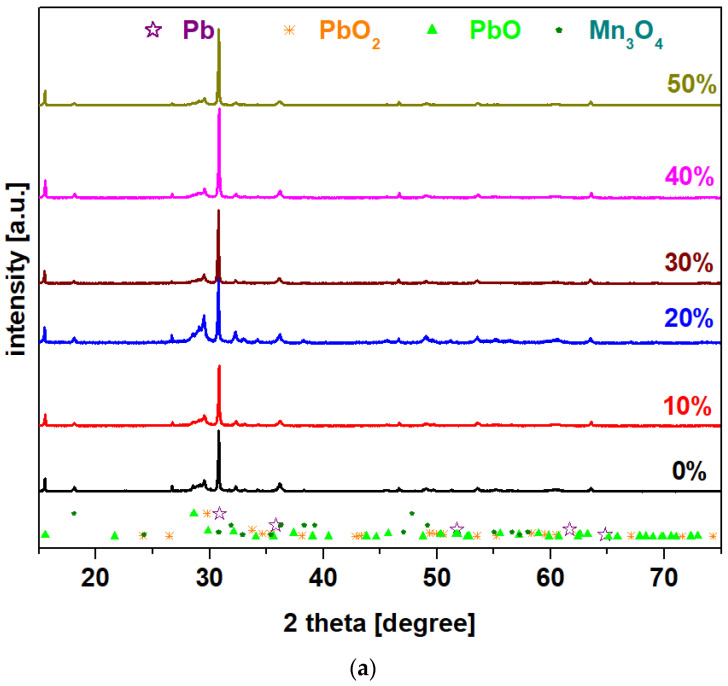

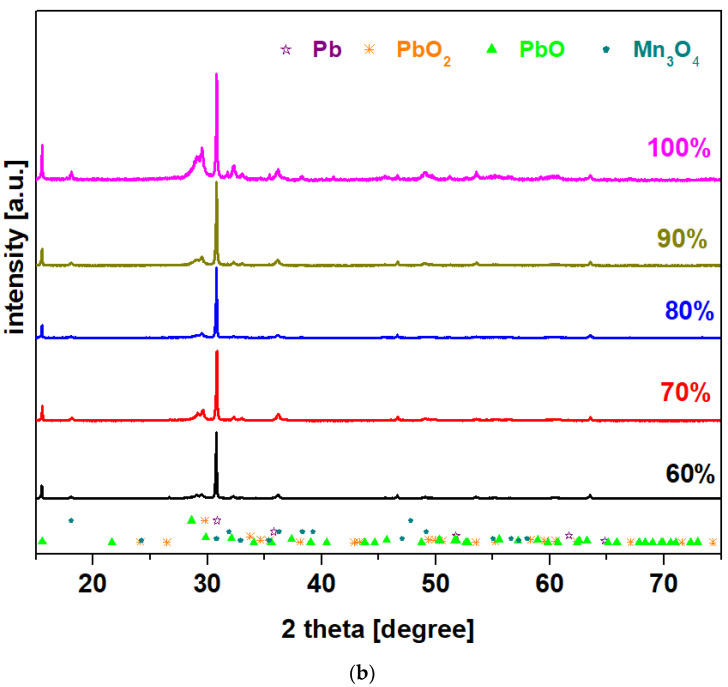
X-ray diffractograms for the vitroceramic system with the chemical formula 15MnO_2_·85[(100 − x)PbO_2_·xPb] where x = 0–100 mol% Pb: (**a**) x = 0–50 mol% Pb; (**b**) x = 60–100 mol% Pb.

X-ray diffractograms of the prepared system with the chemical formula 15MnO_2_·85[(100 − x)PbO_2_·xPb] in which x = 0–100 mol% Pb are presented in Figure 1. For all samples, X-ray patterns reveal that the vitroceramic structures have four crystalline phases: Pb (cubic structure), PbO_2_, PbO, and Mn_3_O_4_ (tetragonal structure). The main diffraction peaks attributed to the PbO and PbO_2_ crystalline phases with orthorhombic structures are those at 2 theta values of 29.08° and 30.08°, respectively. The intensity of the peaks attributed to the PbO and PbO_2_ crystalline phases is increased by doping with high lead content, of up to x = 100 mol% in the host network.

The intensity of the diffraction peaks located at 29.08° and 30.08° increases for the samples with x = 20 and 100 mol% Pb, which indicates the enrichment of the crystalline phases of PbO and PbO_2_. The shifting of the diffraction peaks to lower diffraction angles confirms a weak incorporation of manganese and lead ions into the lead-based matrix. The modifications of the cell volume and other crystallographic parameters are responsible for this shifting to higher/smaller angles because it creates stress in the host matrix. The stress is dependent on the ionic radius of the element [10]. For all samples, the shifting of peaks towards lower diffraction angles is due to the atomic radius of Pb, which is larger than ionic radius of Pb^+4^, Pb^+2^, Mn^+2^, and Mn^+4^ ions.

### 3.2. XANES and EXAFS Data

**Figure 2:**
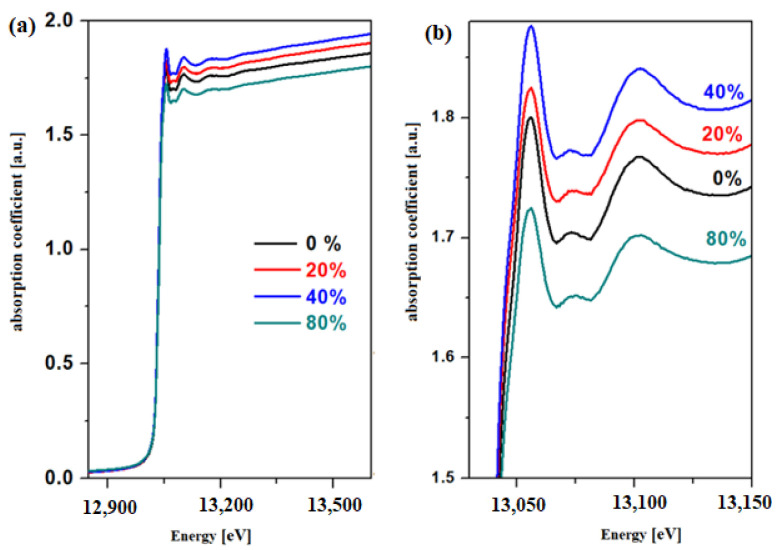
XANES spectra for the L3 edge of the lead atom for the vitroceramic system with chemical formula 15MnO_2_·85[(100 − x)PbO_2_·xPb] where x = 0–80 mol% Pb in the region between (**a**) 12,825 and 13,575 eV (**b**) 13,025 and 13,150 eV.

XANES spectra for the L3 edge of the lead for vitroceramics with the chemical formula 15MnO_2_·85[(100 − x)PbO_2_·xPb] where x = 0, 20, 40, and 80 mol% Pb are represented in Figure 2.

**Figure 3:**
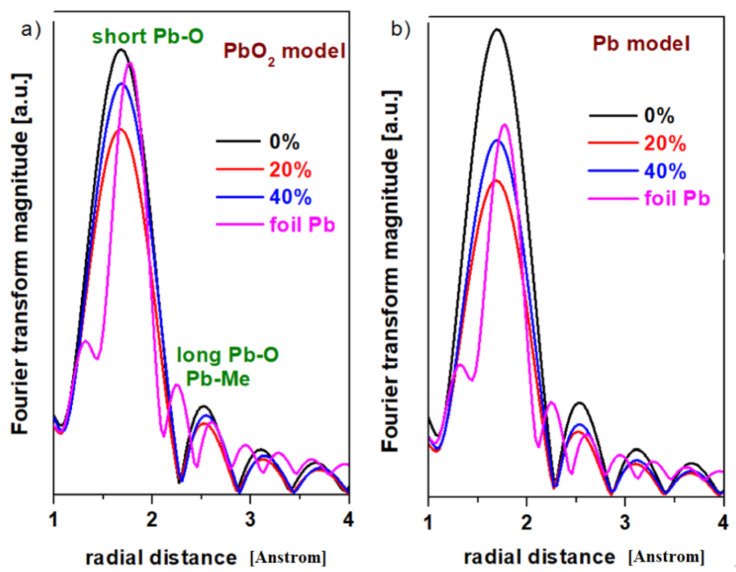
The radial distribution function of the lead atom for the vitroceramic system with chemical formula 15MnO_2_·85[(100 − x)PbO_2_·xPb] where x = 0–40 mol% Pb. (**a**) the PbO_2_ model (**b**) the Pb model.

The analysis of the XANES spectrum indicates that the intensity of the absorption bands changes with the variation in the Pb concentration of the host matrix. This structural evolution suggests variations in the oxidation number of the lead. The EXAFS analysis offers information regarding the local coordination geometry (coordination number) and the distances between the neighboring atoms. In this paper, two theoretical models, namely the PbO_2_ and Pb models, will be tested in the EXAFS simulation with the Artemis program for the prepared samples. The radial distribution functions of the lead atom for the vitroceramics with the chemical formula 15MnO_2_·85[(100 − x)PbO_2_·xPb] formula with x = 0, 20, and 40 mol% Pb are indicated in Figure 3.

The first EXAFS oscillation centered at about 1.6 Å and the second oscillation at 2.5 Å without phase correction correspond to the contributions from Pb-O and Pb-O-Pb respectively [11,12]. The lower intensity of the second EXAFS oscillation and the attenuation of the peaks (oscillations) at greater distances indicate that the samples are disordered at the nanometer scale.

The inspection of the first EXAFS oscillation shows modifications in intensity by doping with higher Pb levels, which suggests that the average oxidation number of the lead atoms was changed.

Table 1 summarizes the parameters for the local structure of the studied vitroceramics: the coordination number, N; the interatomic Pb-O distances; the Debye–Waller parameter of thermal disorder, σ^2^; and the binding energy, E_0_. It is noted that the Debye–Waller parameter of thermal disorder is less than 0.0013. For the simulation with the theoretical model of PbO_2_, it is noticed that the disorder parameter decreases by increasing the lead content, while it was increased for the model of Pb. The disorder parameter, σ^2^, becomes larger with increasing link length or coordination number.

In both models, the smallest values of the Pb-O interatomic distances were obtained for the sample with x = 20% Pb. This implies a higher degree of disorder for the samples with x = 0 and 40 mol% Pb.

### 3.3. Cyclic Voltammetry Measurements

**Figure 4:**
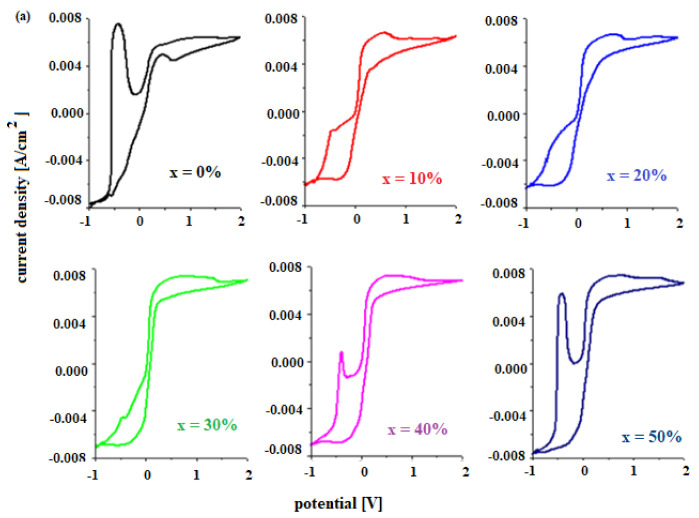

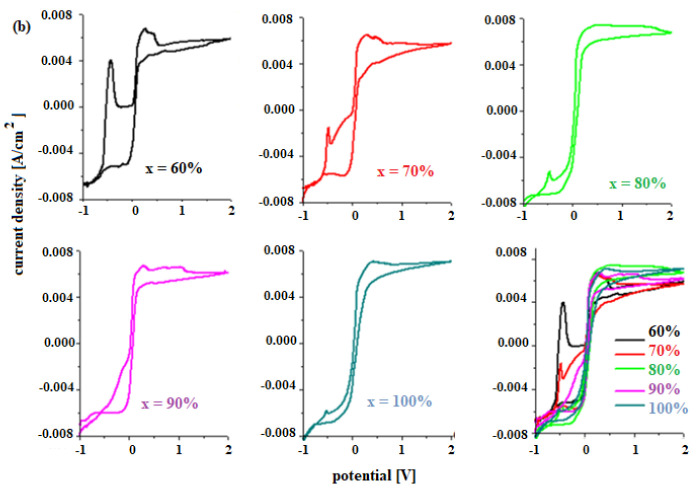
Cyclic voltammograms of the vitroceramics with chemical formula 15MnO_2_·85[(100 − x)PbO_2_·xPb] where (**a**) x = 0–50 mol% Pb and (**b**) x = 60–100 mol% Pb.

The electrochemical properties of materials for use as a car battery electrodes can be determined by voltammetric studies. Figure 4 shows the cyclic voltammograms of the electrode vitroceramics prepared with the chemical formula 15MnO_2_·85[(100 − x)PbO_2_·xPb] with x = 0–100 mol% Pb.

**Figure 5:**
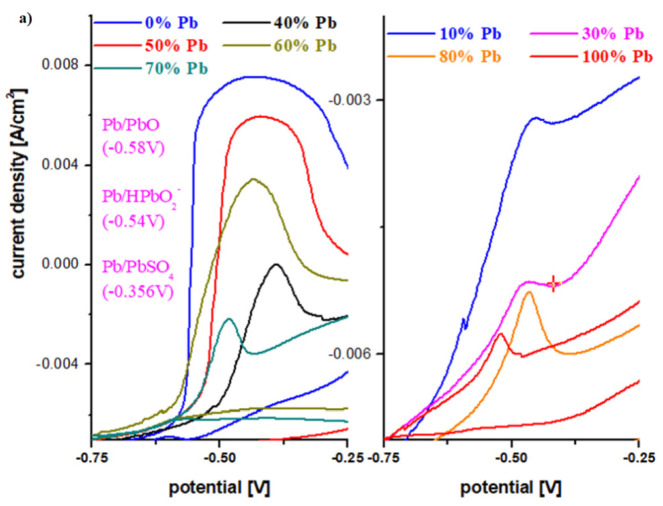

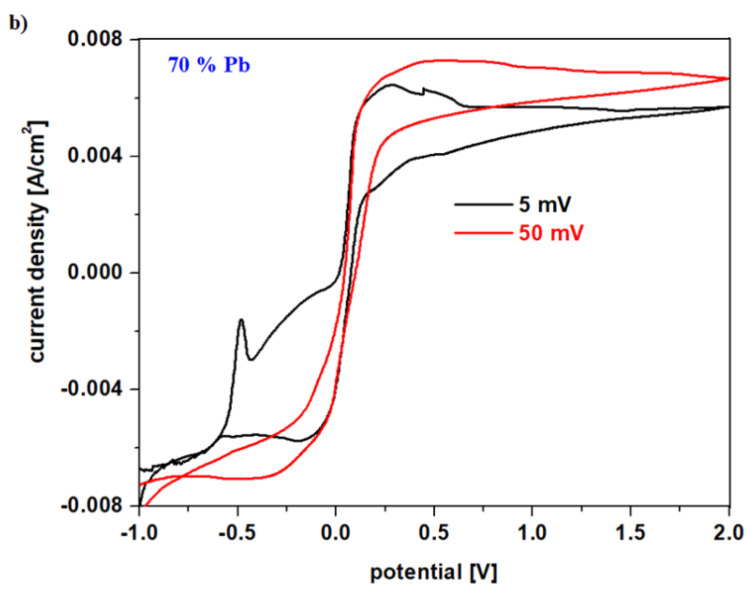
(**a**) First oxidation peak in cyclic voltammograms for the vitroceramic system with chemical formula of 15MnO_2_·85[(100 − x)PbO_2_·xPb] where x = 0–100 mol% Pb in the potential range between −0.75 and −0.25 V; (**b**) Cyclic voltammograms for the vitroceramic system with chemical formula of 15MnO_2_·85[(100 − x)PbO_2_·xPb] where x = 70 mol% Pb at different scan rates.

To describe the first oxidation peak in the anodic region, the cyclic voltammograms in the potential range −0.75 V and −0.25 V are presented in Figure 5a. For the undoped sample, the oxidation peak appears centered at −0.46 V and has the highest intensity. This peak corresponds to an overlap of waves from the following redox systems: Pb/PbO (peak at about −0.58 V), Pb/HPbO_2_^−^ (−0.54 V) and Pb/PbSO_4_ (−0.356 V) [13,14].

The existence of these redox processes implies the formation of a layer of PbO, HPbO_2_^−^, and PbSO_4_ in the cathodic region, which minimizes the performance of the electrode. By adding Pb content in the vitroceramic structure, the intensity of the anodic peaks decreases. For % Pb, the descending order is: 50% > 60% > 40% > 70% > 80% > 10% > 30% > 100% Pb. The lower intensities of the anodic peaks were observed for the samples with x = 10, 20, 30, 90, and 100% Pb.

In the area with positive current density, there are low-intensity oxidation peaks centered at +0.28 V, +1.22 V, and 1.60 V that correspond to the following redox processes PbO_2_/Pb (+0.28 V), O_2_/H_2_O (1.22 V), PbO_2_/Pb (1.455 V), and Mn^+3^/Mn^+2^ (1.54 V). These peaks are responsible for the improved current density in the potentials situated between 0 and 2 V.

The reduction peaks located at about 0.28 V, −0.126 V, and −0.345 V are well defined for the sample with x = 60 mol% Pb. This trend in redox processes suggests that thee redox couples do not produce completely different reactions.

Figure 5b shows the cyclic voltammogram for the vitroceramic with x = 70 mol% Pb scanned at different scan rates. For the scanning speed of 50 mV, no oxidation and reduction waves are detected, while for a scanning speed ten times lower (5 mV) the redox processes that are involved in the production of electric current can be highlighted.

**Figure 6:**
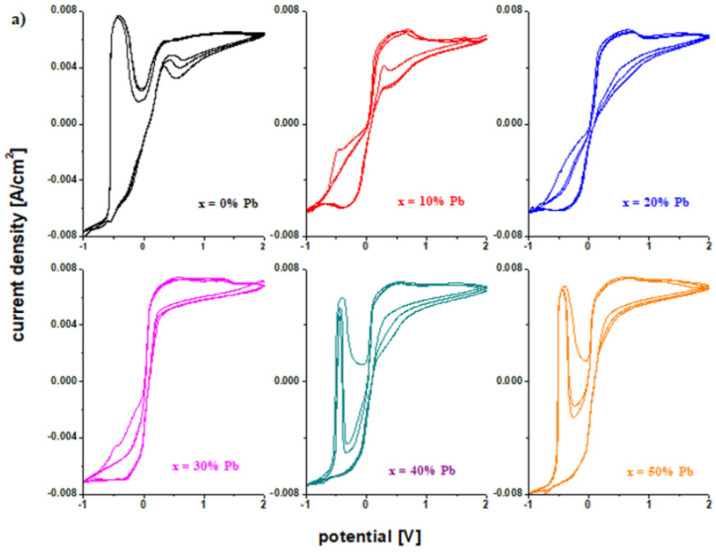

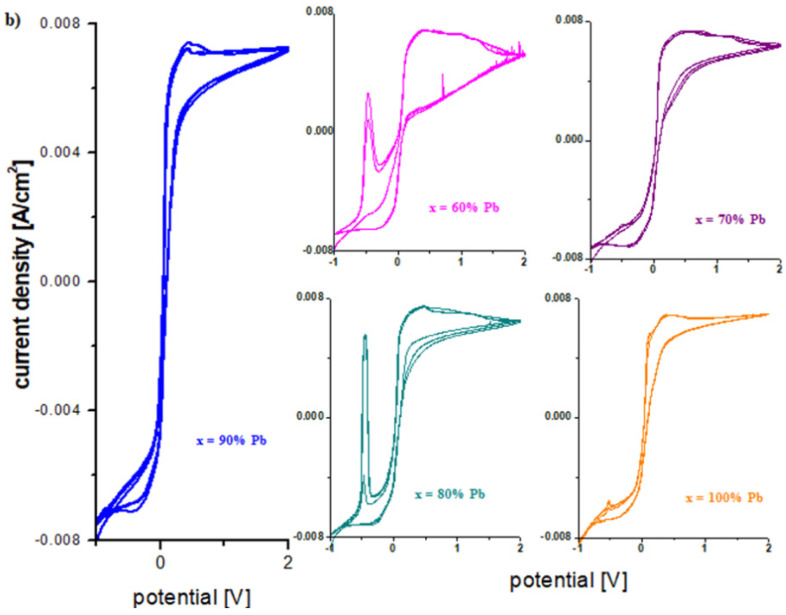
Cyclic voltammograms scanned after three cycles for the vitroceramic system with 15MnO_2_·85[(100 − x)PbO_2_·xPb] chemical formula where (**a**) x = 0–50 mol% Pb and (**b**) x = 60–100 mol% Pb.

The Figure 6 presents the cyclic voltammograms scanned after three cycles for the electrode vitroceramics with the chemical formula of 15MnO_2_·85[(100 − x)PbO_2_·xPb] with x = 0–100 mol% Pb. These graphs are used to verify the reversibility of the redox processes. The cyclic voltammograms for the vitroceramics with x = 0, 40, 50, 60, and 80 mol% Pb indicate a high irreversibility.

After scanning the third cycle (Figure 6), there are changes in the intensity and position of the peak centered at −0.46 V. The shifting of the peak is responsible for the irreversible processes [15]. This process is due to the formation of a PbO and PbSO_4_ layer in the cathodic region. Lead oxide (II) is reduced to metallic lead. The formation of the lead hydroxides will change the concentration of the sulfuric acid solution and, as a result, dimerization reactions occur for the sulfate ions (+2 V). The process of dissolving of the lead sulfate becomes difficult and the electrode loses its efficiency.

For samples with x = 10, 20, 30, 70, 90, and 100% Pb, the voltammograms have a lower degree of irreversibility due to the fact that the intensity of the peaks corresponding to hydroxide and lead (II) oxide is lower.

**Figure 7:**
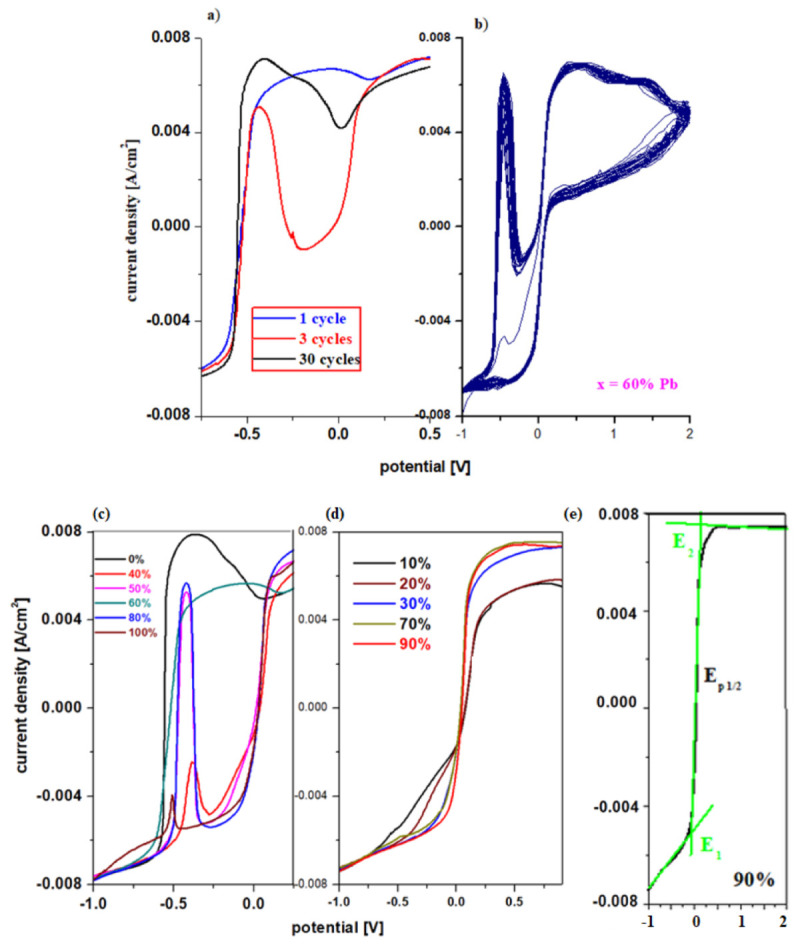
Cyclic voltammograms scanned (**a**) after 1 and 3 cycles and (**b**) after 30 cycles for the vitroceramic system with chemical formula of 15MnO_2_·85[(100 − x)PbO_2_·xPb] where x = 60 mol% Pb. (**c**–**e**) Plots of current density versus potential of linear sweep voltammograms for the vitroceramic system with chemical formula of 15MnO_2_·85[(100 − x)PbO_2_·xPb] where x = 0–100 mol% Pb.

Figure 7a,b shows the cyclic voltammogram scanned after 30 cycles for the sample with x = 60 mol% Pb and the linear sweep voltammograms scanned after the first cycle, 3 cycles, and 30 operating cycles of the working electrode in a 5 M sulfuric acid solution. By increasing the number of scans, the intensities of the peaks centered at −0.55 V and 1.22 V increase.

The cause of the irreversibility of the cyclic voltammogram is the formation of PbO, HPbO_2_^−^, H_2_O, and the dimerization of sulfate ions. After 30 cycles, the intensity of the peak centered at −0.345 V assigned to the formation of lead sulphate was enriched. The presence of the peak at +0.28 V suggests that a part of the formed PbO oxidizes to PbO_2_ and a significant part reacts with the electrolyte solution to form the lead sulfate.

After 30 cycles, the intensity of the current density centered at 0.55 V assigned to the redox systems Pb/PbO (−0.58 V), Pb/HPbO_2_^−^ (−0.54 V) increases, which indicates the accumulation of a layer of lead (II) oxide and its hydroxides to the surface of the electrode. The conversion of lead content into lead (II) oxide produces the following chemical reaction:

PbO + H_2_SO_4_ → PbSO_4_ + H_2_O

and as a result the concentration of the electrolyte solution changes due to the increase in lead sulphate and the dimerization of the sulphate ions (due to the redox system S_2_O_8_^2−^/2SO_4_^2−^ from +2 V). Therefore, there is an advanced process of sulfating of the electrode by increasing the lead sulfate content and the dimerization of the sulfate ions.

### 3.4. Linear Sweep Voltammetry Measurements

The plots of current density versus potential for the linear sweep voltammograms of the vitroceramics in the 15MnO_2_·85[(100 − x)PbO_2_·xPb] chemical formula with x = 0–100 mol% Pb are shown in Figure 7c,d. From the graphical representation of the linear sweep voltammograms for the first anodic peak centered at about −0.55 V can be observed that the intensity for the current density has maximum values for the samples with x = 0, 50, 60 and 80% Pb and minimum values for the samples with x = 40 and 100% Pb. The higher intensity of this oxidation peak produced an higher degree of deterioration (sulfation process) of the studied material used as a grill or electrode in the car battery.

Linear sweep voltammograms for electrode materials with x = 10, 20, 30, 70, and 90 mol% Pb presented in Figure 7c,d show that the anodic peak centered at −0.55 V is below the detection limit of the device. The electrochemical parameters corresponding to the voltammetric response of the electrode, namely the formal potential, E_0_, and the maximum current density intensity, are presented in Table 2.

The highest values of the formal potential and the lowest values of the current density intensity are obtained for electrode materials with a lower Pb content, specifically for x ≤ 30 mol% Pb. The comparative analysis of the results obtained from the voltammetric measurements recommends the samples with x = 70 and 90 mol% Pb as suitable for electrode/grid in the lead accumulator. Our data show that the sample with x = 90 mol% Pb has a slightly better reversibility than that with x = 70 mol% Pb because the value of the formal potential is lower.

### 3.5. Investigation of the Mechanical Properties from Vickers Hardness Measurements

Materials prepared and analyzed as working electrode in the electrolyte solution (5M H_2_SO_4_) were investigated to determine their Vickers hardness [16]. This measurement is used to evaluate the defects or micro-cracks occurring after the introduction of electrode materials into the electrolyte solution. Micro- and macro-cracks influence mechanical performances and the life cycle of the electrode.

**Figure 8:**
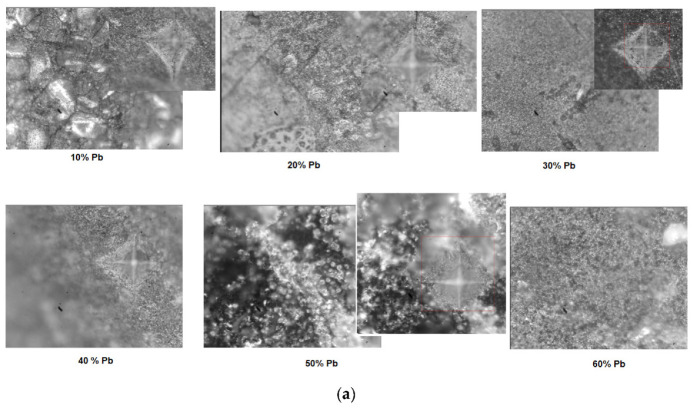

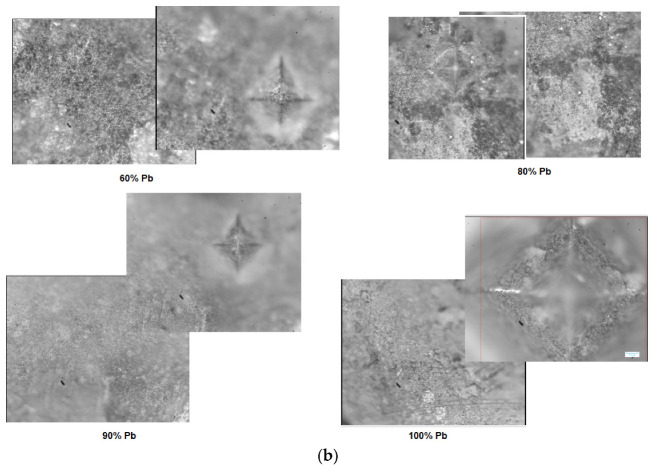

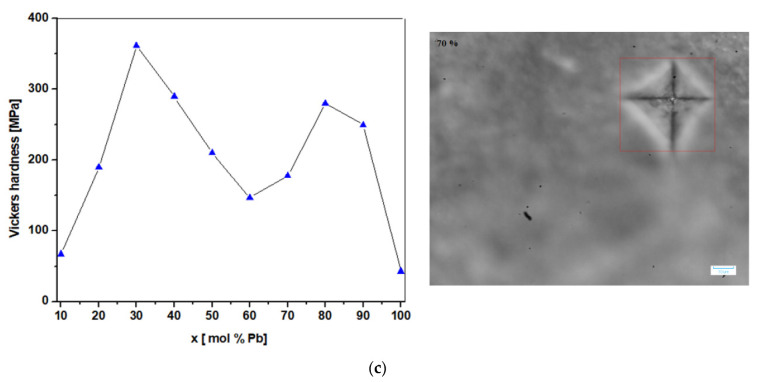
(**a**,**b**) Microscopic images and traces with the pyramidal contour obtained after indentation (the caption with red square represents measured area in the four points) for the vitroceramic system with chemical formula of 15MnO_2_·85[(100 − x)PbO_2_·xPb] where x = 0–100 mol% Pb. (**c**) Compositional dependence of Vickers hardness, HV, for the vitroceramic system with chemical formula of 15MnO_2_·85[(100 − x)PbO_2_·xPb] where x = 0–100 mol% Pb for a force of 0.3 kgf, applied for 15 s.

After indentation, microscopic images with the structures of the electrode materials (after removal from 5M sulfuric acid solution) and traces of pyramidal contour are presented in Figure 8a). For samples with x = 10 and 20 mol% Pb, the microscopic analysis reveals heterogeneous areas and micro-cracks. For samples with x = 30, 40, and 90 mol% Pb some cracks are apparent in the images. For the samples with x ≥ 60 mol%, the surfaces at the microscopic level are heterogeneous and have structural defects.

The graphical representation of the Vickers hardness values according to the sample composition are highlighted in Figure 8b. By comparing the values of Vickers hardness for the studied electrode materials, it was found out that their values increase up to x = 30 mol% Pb, decrease linearly for 40 ≤ x ≤ 60 mol% Pb, then pass again through a maximum for the vitroceramic with x = 80 mol% Pb, after which they decrease again for contents higher than 90 mol% Pb.

The smallest values of the Vickers hardness are found in the samples with x = 10 (HV = 67.2 MPa) and 100 mol% Pb (HV = 42.8 MPa). The sample with x = 20 mol% Pb (HV = 190 MPa) has a slightly lower hardness than the one with x = 90 mol% Pb (HV = 250 MPa), although it has a higher lead content in the structure. The presence of cracks in a vitroceramic affects its mechanical properties, yielding a decrease of Vickers hardness.

Our results show that the presence of micro- and macro-cracks in the studied material produces a decline of the hardness values. The wear process of the electrode vitroceramic is accentuated when the mechanical strength are reduced.

The evolution of the mechanical performances can be associated with the water bounded to material components [17] and with ion mobility, respectively [18]. In the cyclic voltammetry, the presence of the anodic wave situated at about +1.23 V can be correlated with the presence of the water at surface, after the redox reaction:

2H_2_O ↔ O_2_ + 4 H^+^ + 4e^−^

For samples with 30 and 80 mol% Pb, the wave is more present while for the samples with x = 10, 40, 50, 60, 70, 90, and 100 mol% Pb, the wave disappeared. The presence of water at the surface can be explained considering that the material components are mobile in the vitroceramics with x = 30 and 80 mol% Pb and result in an increase of the Vickers hardness value. For vitroceramics with x = 10, 60, and 100 mol% Pb, the smaller values of the Vickers hardness can be associated with the rigidity of the components in the host matrix.

The sample with x = 90 mol% Pb has the lowest value of the formal potential which suggests a good reversibility of cyclic voltammogram (according to Table 2). In addition, the higher value of the current density intensity (Table 2) and Vickers hardness (Figure 8c) can be observed for the sample with 90 mol% Pb. These parameters indicate the good electrochemical and mechanical performance for this sample.

## 4. Conclusions

In this paper, the structure and electrochemical and mechanical performances of the vitroceramic system with the chemical formula of 15MnO_2_·85[(100 − x)PbO_2_·xPb] with x = 0–100% mol Pb were reported. The XRD data indicate the occurrence of vitroceramic structures for all samples.

XANES data indicate that the local structure around Pb atoms has a varied compositional dependence. The analysis of EXAFS data indicates variation in the Pb-O interatomic distances in the first coordination sphere for the studied vitroceramics.

The electrode vitroceramics were subjected to voltammetric and hardness measurements. Our results indicate the sample with x = 90 mol% Pb as most suitable for electrode vitroceramic in car battery applications, both in terms of microstructure (trace indentation did not lead to cracks) as well as of electrochemical properties (high current density intensity, low formal potential, and good reversibility of the cyclic voltammogram).

## Figures and Tables

**Table 1:** Parameters of the global structure for the lead atom in the first coordination sphere for the vitroceramics in the system with chemical formula 15MnO_2_·85[(100 − x)PbO_2_·xPb] with x = 0, 20 and 40 mol% Pb.

Theoretical Model Used in Simulation—PbO_2_				
Investigated Sample	Coordination Number, N_1_	Interatomic Pb-O Distance R_1_ ± ΔR_1_ [Å]	Debye–Waller Parameter of Thermal Disorder (σ^2^)	Binding Energy, E_0_ [eV]
x = 0	4	2.26211	0.00130	13,039.569
x = 20	4	2.25457	0.00083	13,038.25
x = 40	4	2.26783	0.00075	13,038.992
**Theoretical Model Used in Simulation—Pb**				
x = 0	4	2.26831	0.00039	13,039.569
x = 20	4	2.25726	0.00084	13,038.25
x = 40	4	2.26774	0.00082	13,038.992

**Table 2:** Electrochemical parameters corresponding to the linear sweep voltammetric response of the electrode material, namely the formal potential, E_0_, and the maximum current density intensity.

Composition of the Electrode Material, x [mol% Pb]	Formal Potential, E_0_ [V]	Maximum Intensity of Current Density [A/cm^2^]
10%	0.0596	0.00691
20%	0.0586	0.00678
30%	0.0514	0.00735
70%	0.0489	0.00756
90%	0.04415	0.00750

## References

[1] Rada S., Rus L., Rada M., Zagrai M., Culea E., Rusu T. (2014). Compositional dependence of structure, optical and electrochemical properties of antimony (III) oxide doped lead glasses and vitroceramics. Ceram. Int..

[2] Rada S., Zagrai M., Rada M., Magerusan L., Popa A., Suciu R., Macavei S., Suciu M. (2018). Structure, electrochemical characterizations and the role of copper oxide in lead-lead dioxide glasses and vitroceramics. J. Non-Cryst. Solids.

[3] Rada M., Popa A., Rada S., Bot A., Culea E. (2019). Recycled and vanadium-doped materials as negative electrode of the lead acid battery. J. Solid State Electrochem..

[4] Rada M., Zagrai M., Rada S., Bot A., Culea E. (2017). Effects on the characteristics of bonding and local structure in molybdenum-lead-lead dioxide glasses and vitroceramics. J. Alloys Compd..

[5] Rada S., Unguresan M., Rada M., Cuibus D., Zhang J., Pengfei A., Suciu R., Bot A., Culea E. (2019). Manganese-lead-lead dioxide glass ceramics as electrode materials. J. Electrochem. Soc..

[6] Rada S., Cuibus D., Vermesan H., Rada M., Culea E. (2018). Structural and electrochemical properties of recycled active electrodes from spent lead acid battery and modified with different manganese dioxide contents. Electrochim. Acta.

[7] Abouhaswa A.S., Rammah Y.S., Sayyed M.I., Tekin H.O. (2010). Synthesis, structure, optica land gamma radiation shielding properties of B_2_O_3_-PbO_2_-Bi_2_O_3_ glasses. Compos. Part B.

[8] Chromcikova M., Svoboda R., Hruska B., Osipov A.A., Osipova L.M., Pecusova B., Nowicka A., Liska M. (2022). Thermokinetic behavior of the Al2O3-PbO-B2O3 glasses. J. Non-Cryst. Solids.

[9] Zhong C., Yan J., Jiang Q., Chen C., Yuan S., Zeng H., Du J. (2022). Experimental characterizations and molecular dynamics simulations of the structures of lead aluminosilicate glasses. J. Non-Cryst. Solids.

[10] Redhu P., Punia R., Hooda A., Malik B.P., Sharma G., Sharma P. (2020). Correlation between multifunctional properties of lead free ion doped BCT perovskite ceramics. Ceram. Intenational.

[11] Dehelean A., Rada S., Zhang J. (2020). Determination of the lead environment in samarium-lead oxide-borate glasses and vitroceramics using XANES and EXAFS studies. Radiat. Phys. Chem..

[12] Yu Y.H., Tyliszczak T., Hitchcock A.P. (1990). Pb L3 EXAFS and Near-Edge studies of lead metal and lead oxides. J. Phys. Chem. Solids.

[13] Rada S., Unguresan M., Rada M., Tudoran C., Wang J., Culea E. (2020). Performance of the recycled and copper-doped materials from spent electrodes by XPS and Voltammetric characteristics. J. Electrochem. Soc..

[14] Rada S., Unguresan M.L., Bolundut L., Rada M., Vermesan H., Pica M., Culea E. (2016). Structural and electrochemical investigations of the electrodes obtained by recycling of lead acid batteries. J. Electroanal. Chem..

[15] Meng X., Zhao H., Bi S., Ju Z., Yang Z., Yang Y., Li H., Liang J. (2022). Electrochemical mechanism of molten electrolysis from TiO_2_ to titanium. Materials.

[16] Vasiliu R.D., Utu I.D., Rusu L., Bolos A., Porojan L. (2022). Fractographic and microhardness evaluation of all–ceramic hot–pressed and CAD / CAM restorations after hydrothermal aging. Materials.

[17] Florea I., Jumate E., Manea D.L., Fechete R. (2019). NMR study on new natural building materials. Procedia Manuf..

[18] Zhang E., Fulik N., Paasch S., Borchardt L., Kaskel S., Brunner E. (2019). Ionic liquid—Electrode materials interactions studied by NMR spectroscopy, cyclic voltammetry, and impedance spectroscopy. Energy Storage Mater..

